# Cryopreservation in a Standard Freezer: −28 °C as Alternative Storage Temperature for Amniotic Membrane Transplantation

**DOI:** 10.3390/jcm11041109

**Published:** 2022-02-19

**Authors:** Joana Witt, Luis Grumm, Sabine Salla, Gerd Geerling, Johannes Menzel-Severing

**Affiliations:** 1 Department of Ophthalmology, Medical Faculty, Heinrich-Heine-University of Düsseldorf, 40225 Düsseldorf, Germany; joana.witt@med.uni-duesseldorf.de (J.W.); luis.grumm@med.uni-duesseldorf.de (L.G.); geerling@med.uni-duesseldorf.de (G.G.); 2 Department of Ophthalmology, RWTH Aachen University, 52074 Aachen, Germany; ssalla@ukaachen.de

**Keywords:** amniotic membrane, cryopreservation, appropriate technology, international ophthalmology

## Abstract

Human amniotic membrane (hAM) is usually stored at −80 °C. However, in many regions, cryopreservation at −80 °C is not feasible, making hAM unavailable. Therefore, the possibility of cryopreservation at −28 °C (household freezer) was investigated. hAMs (*n* = 8) were stored at −80 °C or −28 °C for a mean time of 8.2 months. hAM thickness, epithelial integrity and basement membrane were assessed histologically. The collagen content, concentration of hepatocyte growth factor (HGF) and basic fibroblast growth factor (bFGF) were determined. Elastic modulus and tensile strength were measured. The mean thickness of hAM stored at −28 °C was 33.1 ± 21.6 µm (range 9.7–74.9); thickness at −80 °C was 30.8 ± 14.7 µm (range 13.1–50.7; p = 0.72). Mean collagen content, epithelial cell number and integrity score showed no significant difference between samples stored at −28 °C or −80 °C. Basement membrane proteins were well preserved in both groups. Mean tensile strength and elastic modulus were not significantly different. Concentration of bFGF at −28 °C was 1063.2 ± 680.3 pg/g (range 369.2–2534.2), and 1312.1 ± 778.2 pg/g (range 496.2–2442.7) at −80 °C (*p* = 0.11). HGF was 5322.0 ± 2729.3 pg/g (range 603.3–9149.8) at −28 °C, and 11338.5 ± 6121.8 pg/g (range 4143.5 to 19806.7) at −80 °C (*p* = 0.02). No microbiological contamination was detected in any sample. The cryopreservation of hAM at −28 °C has no overt disadvantages compared to −80 °C; the essential characteristics of hAM are preserved. This temperature could be used in an alternative storage method whenever storage at −80 °C is unavailable.

## 1. Introduction

Human amniotic membrane (hAM) is the innermost layer of the placenta that surrounds the fetus. It is composed of an epithelial monolayer, an underlying basement membrane and an avascular stroma [1]. Transplantation of hAM in ophthalmology was first introduced by de Rotth in 1940 [2] for ocular surface reconstruction in patients with symblepharon but was widely unrecognized until its reintroduction and establishment by Kim and Tseng in 1995 [3]. Due to its many beneficial properties, hAM has since then been increasingly used for a variety of ocular pathologies including persistent corneal ulcerations, conjunctival lesions or corneal disorders associated with limbal stem cell deficiency [4,5,6,7].

hAM contains several growth factors and cytokines and has the ability to promote epithelialization (e.g., in case of persistent corneal epithelial defects) and to reduce inflammation and fibrosis [8,9,10,11,12]. Furthermore, hAM has anti-microbial [13], pro- or anti-angiogenic [14] and immunomodulatory [15] properties. Immunosuppressive treatment in allogeneic hAM transplantation is thus not necessary.

Depending on the respective pathology, different surgical techniques have been established for the application of hAM. It can be used as a graft (inlay) to act as a substrate or basement membrane for host epithelial cells or as a patch (overlay) protecting the host epithelium so that re-epithelialization can occur underneath. A combination of both techniques is also frequently applied [5,16].

hAM is prepared from human placentas obtained by elective cesarean section and is subsequently washed and prepared under sterile conditions. Although unprocessed and unpreserved (“fresh”) hAM has been used in some studies [17,18], legal regulations in most countries require that it must be quarantined (i.e., stored) prior to transplantation to allow for testing of hAM sterility and serological testing of the donor [19]. The transplant can be stored for up to 24 months, which significantly increases its accessibility [20].

Cryopreservation is the most commonly used method for the storage of hAM [3,6,8,20]. However, this method requires an ultra-deep-freezing facility (to −80 °C), which is expensive to buy and maintain. For these reasons, freezing at −80 °C is frequently unavailable, for example in (but not limited to) countries of the Global South [21], despite the fact that the beneficial effects of hAM in ophthalmology have been consistently recognized, and its use has been suggested as beneficial [22] and cost-effective [23] for developing countries. In other fields, researchers have successfully implemented low-cost adaptations of advanced medical procedures that required cryopreservation to match local prerequisites, such as stem cell transplantation for neuroblastoma [24]. Hence, we postulate that if storage at −28 °C (using a standard household freezer) could be shown to preserve clinically relevant properties of hAM in the same way as storage at −80 °C can, this may be beneficial particularly for regions with a high incidence of ocular surface diseases that are currently not using hAM in their clinical routine.

In this study, we therefore investigated the effects of −28 °C (standard freezer) and −80 °C cryopreservation on the morphological, mechanical and biological properties of hAM that are deemed relevant for its therapeutic actions.

## 2. Materials and Methods

hAMs were provided by the Cornea Bank Aachen. All donors were tested to exclude an infection with hepatitis virus type B and C, human immunodeficiency virus and syphilis. hAMs were prepared as described in [25]; this protocol corresponds exactly to the standard routine in this eye bank and has been approved for clinical use by the relevant local and federal authorities. In brief, human placentae were obtained after elective cesarean sections from healthy women. Placentae were dissected under sterile conditions and rinsed twice with 500 mL of isotonic solution (Ringer, B. Braun, Melsungen, Germany) without any cell culture medium, glycerol, antibiotics or other additives. hAMs were carefully separated from chorion and placed onto a carrier membrane (Raucocel, Lohmann and Rauscher, Rengsdorf, Germany) that was cut into pieces of 3.75 cm × 3.75 cm. 

Two hAM samples of each of the eight different donors were processed. The mean donor age was 33 ± 4 years (Table 1). The pieces were transferred to 50 mL centrifuge tubes (Falcon, Corning, New York City, NY, USA) and stored without any medium or additives (“straight” cryopreservation) for 8.2 ± 2 months (range 7–12). One sample from each donor was frozen at −28 °C (Freezer GN 1056, Liebherr, Bulle, Switzerland) and one at −80 °C (Ultra-low freezer Forma 900, Thermo Fisher Scientific, Waltham, MA, USA). For testing, hAM samples were thawed at room temperature for 30 min.

hAM samples were fixed in 4% paraformaldehyde (RotiHistofix, Roth, Karlsruhe, Germany) for 2 h and paraffin-embedded. Serial sections of 4 μm in thickness were cut using a microtome (Leica RM2255, Leica Biosystems, Wetzlar, Germany) and stained with hematoxylin and eosin (both Roth, Karlsruhe, Germany).

Three sections per hAM sample were analyzed by blinded examiners under a light microscope (Leica DM 4000B, Leica Microsystems, Wetzlar, Germany) using a × 400 magnification. A grading system for epithelial integrity from score 0 to 3 was implemented. Criteria for the rating were the integrity of the epithelium and the arrangement and degeneration (vacuolization, karyopyknosis or karyolysis) of epithelial cells. A score of 0 represents an entirely regular and normal epithelium, whereas a score of 3 means that the epithelium was severely damaged. Examples and criteria of each score are shown in Table 2.

To measure average epithelial thickness and the number of epithelial cells per mm, three sections of each sample were photographed in full length at 200× magnification. Epithelial thickness measurements and cell counting were performed using Fiji’s freehand tool [26].

Immunohistochemical staining was performed for basement membrane proteins laminin, fibronectin and collagen VII. Therefore, paraffin sections were deparaffinized and rehydrated using xylol and ethanol in decreasing concentrations. Heat-based antigen retrieval was used for the staining of laminin and fibronectin. hAM sections were placed in a 10 mM sodium-citrate buffer (pH = 6) at 95 °C for 30 min, followed by 20 min of cooling on ice. Afterward, samples for staining of laminin were placed into 20 µg/mL Proteinase K in TE buffer (50 mM Tris, 1 mM EDTA, 0.5% Triton X-100, pH = 8) for 10 min, whereas for fibronectin, 0.1% Triton was utilized. In the case of collagen VII, antigen retrieval was performed only using 20 µg/mL Proteinase K in TE buffer for 60 min at room temperature. For all three proteins, the sections were then blocked with 5% donkey serum for 30 min. Primary antibodies (anti-laminin rabbit, ab11575; anti-fibronectin mouse, ab6328; anti-collagen VII rabbit, ab93350; all Abcam, Cambridge, UK) were diluted in 2% donkey serum (laminin 1:50, fibronectin 1:50, collagen VII 1:200) and applied overnight at 4 °C. Secondary antibodies anti-mouse Alexa Fluor 488 or anti-rabbit Alexa Fluor 488 (Jackson ImmunoReasearch Biotechnology Co., West Grove, PA, USA) were diluted 1:500 in phosphate-buffered saline (PBS) and applied for 60 min at room temperature. Slides were mounted using Mowiol (Roth, Karlsruhe, Germany) containing 4′,6-diamidino-2-phenylindole (DAPI, Thermo Fisher Scientific, Waltham, USA) to visualize cell nuclei. 

The amount of collagen I, II, III, V and XI in the hAM samples after storage was determined using the Sircol 2000 insoluble Collagen Assay (Biocolor, Carrickfergus, UK). Therefore, samples were thawed and subsequently incubated with 50 µL/mg fragmentation reagent for 3 h at 65 °C. Results were measured as absorption at 530 nm, using a multilabel plate reader (ViktorX, PerkinElmer, Waltham, MA, USA).

hAMs were rinsed with 1 mL PBS and separated from the carrier membrane. Then, samples were cut into pieces of 5 mm × 10 mm and placed in a material testing machine (Zwickiline Z0.5 TN, ZwickRoell, Ulm, Germany) equipped with Vulkollan-coated clamps and a 20 N load. Samples were kept moist during measurements. Samples were strained at a rate of 5 mm/s. Force and elongation were measured until a sudden decrease in force (80%) indicated a rupture or partial rupture of the samples. A stress−strain curve was generated, and tensile strength and elastic modulus (Young’s modulus) were calculated. Tensile strength is defined as the maximum stress that a material can withstand while being stretched before rupturing, while the elastic modulus is a measure of a material’s resistance to elastic, non-permanent deformation when stress is applied to it.

Enzyme-linked immunosorbent assays (ELISA) were performed according to the manufacturer’s protocol to evaluate the amount of hepatocyte growth factor (HGF) and basic fibroblast growth factor (bFGF) (both Elabscience, Houston, TX, USA) in the stored hAM samples. Pieces of hAM were repeatedly snap frozen in liquid nitrogen and grinded using a glass tissue grinder (Wheaton Science Products, Millville, NJ, USA). Afterward, 3 mL PBS per gram hAM sample were added. The supernatants (centrifuged at 20,000× *g* for 1 min) were frozen at −80 °C until analysis. Before performing ELISA, frozen supernatants were thawed on ice.

Microbiological analysis to detect possible contamination was performed after storing and thawing the samples. Therefore, a 25 mm² biopsy of every hAM was incubated in a blood culture system (BD Bactec Plus Aerobic/Anaerobic F Medium, Becton, Dickinson and Company, Franklin Lakes, NJ, USA) under aerobic and anaerobic conditions for 7 days at 30 °C.

Statistical analysis was performed using GraphPad Prism Version 6, (GraphPad Software, San Diego, CA, USA). Results are expressed as mean ± standard deviation. Paired Student’s t-test was used for comparisons between two groups. *p* values ≤ 0.05 were considered statistically significant.

## 3. Results

### 3.1. Integrity of hAM Stroma, Epithelium and Epithelial Basement Membrane

**Figure 1:**
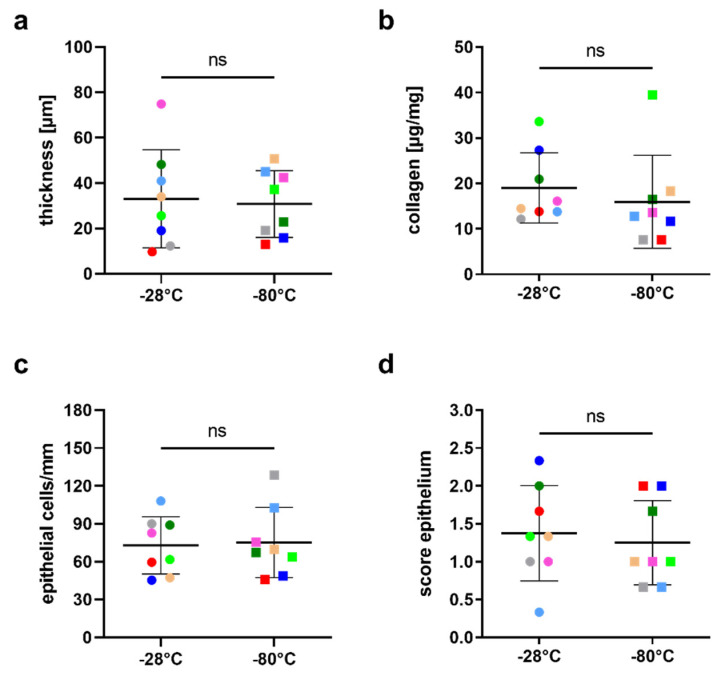
Thickness (**a**), collagen content (**b**), epithelial cell numbers (**c**) and epithelial cell morphology (**d**) after storage at −28 °C and −80 °C, respectively. Each data point represents the average of all three measurements on samples from one donor at the respective temperature. Horizontal bars show mean values for all donors at the respective temperature ± standard deviation. Pairs from the same donor are shown in matching colors. ns: Not statistically significant.

On average, hAMs stored at −28 °C were 33.1 ± 21.6 µm thick (range 9.7 to 74.9) and hAMs stored at −80 °C measured 30.8 ± 14.7 µm (range 13.1 to 50.7; *p* = 0.72) (Figure 1a). No loosening or damage to the hAM stroma was observed at either storage temperature. Collagen, the major structural protein of the hAM stroma, was not reduced by lowering storage temperature to −28 °C compared to storage at −80 °C (Figure 1b). The mean collagen content was 19.0 ± 7.7 µg/mg (range 12.1–33.6) for −28 °C and 16.0 ± 10.2 µg/mg (range 7.6–39.5) for −80 °C (*p* = 0.23).

Results of the quality and integrity scoring of the hAM epithelium are shown in Table 2. In the −28 °C group, 73 ± 23 epithelial cells per mm (range 45–108) were counted in the histological cross-section, whereas for −80 °C, there were 75 ± 28 cells per mm (range 46–129; *p* = 0.75) (Figure 1c). Integrity score showed no significant difference between samples stored at −28 °C (1.38 ± 0.63; range 0.33–2.33) and those stored at −80 °C (1.25 ± 0.56; range 0.67–2.0; *p* = 0.29) (Figure 1d).

**Figure 2:**
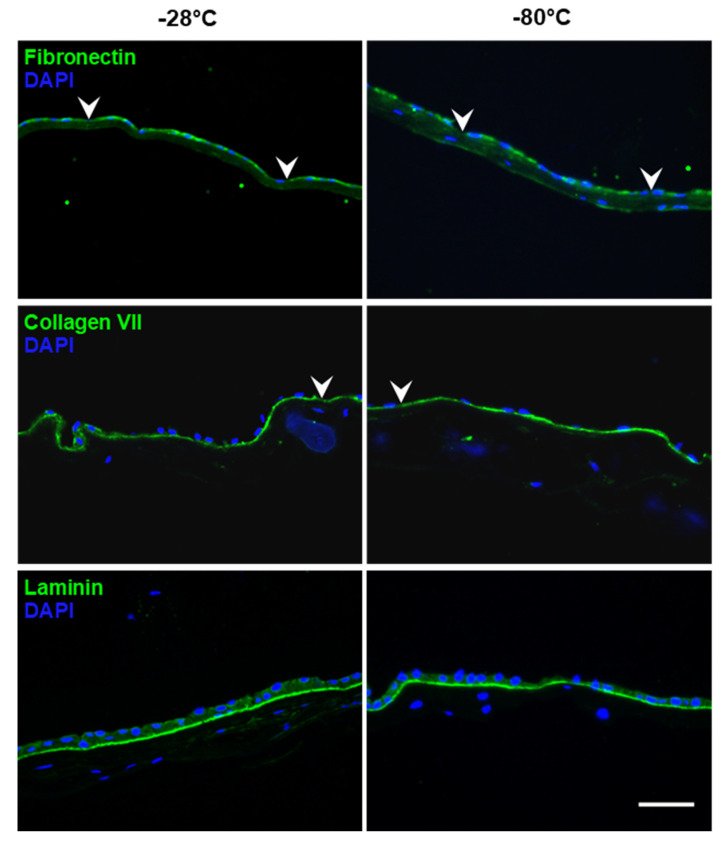
Representative immunohistochemical stains of basement membrane proteins (green) after storage at −28 °C and −80 °C, respectively. Arrowheads: Disruption of staining/basement membrane. DAPI: 4′,6-diamidino-2-phenylindole (nuclear counterstain). Scale bar: 50 µm.

Figure 2 shows representative images of the immunohistological staining of fibronectin, collagen VII and laminin. All three proteins were visible in all analyzed samples and showed a specific binding to the basement membrane of the hAM epithelium (as a line beneath). Fibronectin and collagen VII staining showed some small disruptions in the staining intensity (indicated by arrowheads) of the basement membrane in both −28 °C and −80 °C samples. Fibronectin was also detected in the stroma in both groups. Laminin expression was present as a continuous line in all samples, indicating that the storage temperature did not affect the protein. Negative controls displayed no specific staining.

Overall, hAMs were well preserved in both groups and no storage-temperature-dependent differences were detectable.

### 3.2. Influence of the Storage Temperature on hAM Biomechanics

**Figure 3:**
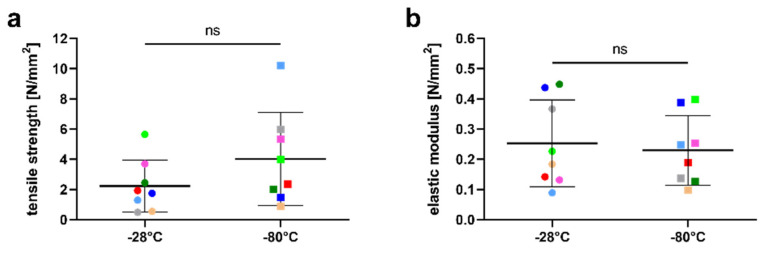
Tensile strength (**a**) and elastic modulus (**b**) after storage at −28 °C and −80 °C, respectively. Horizontal bars show mean values ± standard deviation. Pairs from the same donor are shown in matching colors. ns: Not statistically significant.

The mean tensile strength of hAM stored at −28 °C was 2.23 ± 1.73 N/mm^2^ (range 0.49–5.65. This was slightly lower compared to 4.03 ± 3.09 N/mm^2^ (range 0.88–10.21) at −80 °C; however, the difference was not statistically significant (*p* = 0.2) (Figure 3a). The calculation of the elastic modulus based on the stress−strain curve resulted in 0.25 ± 0.14 N/mm^2^ (range 0.09–0.45) for the −28 °C group; this was also not significantly different from the elastic modulus of 0.23 ± 0.12 N/mm^2^ (range 0.1–0.4) in the −80 °C group (*p* = 0.73) (Figure 3b).

### 3.3. Influence of Storage Temperature on Growth Factor Content 

**Figure 4:**
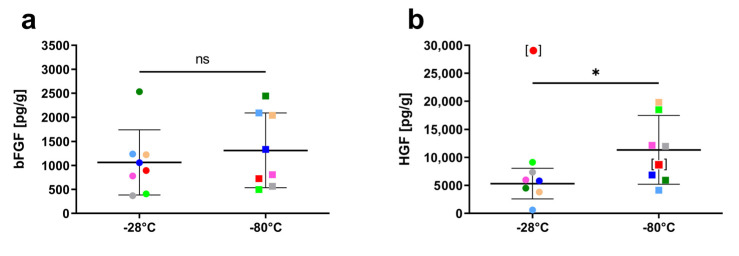
Growth factor levels of basic fibroblast growth factor (bFGF, (**a**)) and hepatocyte growth factor (HGF, (**b**)) after storage at −28 °C and −80 °C, respectively. Horizontal bars show mean values ± standard deviation. Pairs from the same donor are shown in matching colors. ns: Not statistically significant; Asterisk: *p* < 0.05; [ ]: Outlier excluded from statistical analysis.

The content of the growth factors bFGF and HGF in hAM after storage at −28 °C and −80 °C is shown in Figure 4. The average concentration of bFGF in hAM at −28 °C (1063.2 ± 680.3 pg/g; range 369.2–2534.2) was not significantly lower compared to the storage at −80 °C (1312.1 ± 778.2 pg/g; range 496.2–2442.7; *p* = 0.11) (Figure 4a). HGF content was measured as 8303.5 ± 8803.5 pg/g (range 603.3–29174.3) for −28 °C and 10997.5 ± 5749.2 pg/g (range 4143.5–19806.7) for −80 °C, and this difference was also not statistically significant (*p* = 0.49). However, the HGF content of one hAM sample in the −28 °C group (Figure 4b, data point shown in square brackets) was identified as a statistical outlier via Grubb’s method as well as ROUT’s. Analysis excluding the outlier pair led to an average of 5322.0 pg/g HGF (range 603.3–9149.8) in the −28 °C group, which was slightly but significantly lower compared to hAM stored at −80 °C (mean 11338.5 pg/g; *p* = 0.02).

### 3.4. Sterility

No microbial contamination of any kind was found in the examination of all hAM.

## 4. Discussion

Its numerous beneficial properties have made hAM one of the most frequently used tools for a variety of indications in ocular surface reconstruction [5,7,8,10,27,28,29]. Although the precise mechanism of action of hAM is not fully understood, the main clinical benefits seem to reside in the combination of the presence of the basement membrane, the extracellular matrix and growth factors and cytokines within the hAM [12,30,31]. In clinical and eye bank practice, however, apart from checking sterility and a macroscopic visual inspection during preparation, no further examinations take place on the graft before use with regard to tissue quality, transparency, thickness and growth factor content [32]. Accordingly, there are no clear reference values that qualify a hAM for its use as a graft. Therefore, to assess whether hAM preserved using a readily available household freezer meets the same requirements as hAM preserved using the established protocol, we cryopreserved hAM samples at either −28 °C or −80 °C and directly compared their histological and mechanical characteristic and protein levels. For these comparisons, epithelial integrity, epithelial cell count, basement membrane protein expression, tensile strength, elastic modulus and growth factor levels were assessed.

Differences in thickness and hence resistance may affect surgical handling and can lead to inconsistency in clinical outcomes [4,33]. Here, no such difference could be detected. It is important however to note that the size and thus the thickness of tissue samples can vary in histological sections depending on the compression and stretching level during formalin fixation and on the embedding and processing procedure (e.g., fixation and dehydration duration). Other than the method of processing, it has been reported that the sample site of hAM may impact its effects in tissue regeneration; also, inter-donor variations in the physical structure of AM can be related to age, maternal health or gestational age [13,34,35,36]. This may help to explain the variations between donors seen in this and several other studies [26,37,38], particularly because the sub-region each specimen was taken from was not specified. Indeed, across all parameters this study looked at, inter-donor-dependent differences were higher than the storage-temperature-dependent differences. This reflects the clinical reality because all specimens were obtained using an approved, routine protocol.

The integrity of the hAM basement membrane proteins laminin, fibronectin and collagen IV and VII is crucial for applications in ocular surface surgery. The proteins promote epithelial cell migration and adhesion, and the stromal extracellular matrix eases inflammation, scarring and neovascularization [6,8,12,30]. In our study, immunohistochemical examination showed immunoreactivity for laminin, fibronectin and collagen VII. We found no difference between both storage temperatures with regard to the preservation of these basement membrane proteins. They formed an almost continuous line (except for a few, small disruptions) along the basement membrane in all samples in a similar distribution, as reported by other groups [39,40,41,42].

Koizumi et al. have demonstrated that many of the therapeutically relevant growth factors are localized mainly in the hAM epithelium. Hence, their integrity should be preserved after storage [31]. However, differences in hAM preservation procedure can lead to loss of epithelial cells and a higher degree of cellular degeneration [37]. Wagner et al. showed that straight frozen hAM, as was used in our study, did not show any significant impact on epithelial cell number or epithelial integrity compared to fresh controls after 6 months of storage at −80 °C [25]. Our data extend these findings to straight frozen hAM stored at −28 °C, since our score and cell number also did not differ between the 2 temperatures.

Preservation of elasticity and tensile strength is crucial for hAM transplantation, not least because sutures are the usual means of fixating hAM in the host bed [43]. The compact layer of the hAM stroma has the greatest resistance to tensile forces and stresses [21]. The freezing process and the formation of ice crystals can lead to significant changes in tissues. Freezing and thawing lead to osmotic stress and dehydration, which can result in an increase in tensile strength [40]. For example, a study investigating cryopreserved collagen-based blood vessels concluded that another reason for the increase in tensile strength during tissue cryopreservation may be temperature-induced cross-linking of collagens [44]. Accordingly, in their work on cryopreserved hAM, Wagner et al. found an increase in tensile strength with longer storage time (6 months) in straight frozen hAM [25]. Our data do not show significant differences in elastic modulus or tensile strength between the different storage temperatures employed. However, we did observe a tendency toward lower tensile strength when hAM was stored at −28 °C as opposed to −80 °C. This observation may become statistically significant with a larger number of samples, the low number of amniotic membranes used in this study (*n* = 8) being one of its limitations. However, it is likely not clinically meaningful, as other studies have observed much lower tensile strength (e.g., 0.16 ± 0.07 N/mm² [45]; 0.32 ± 0.14 N/mm² [46]; in spite of this, however, hAM was deemed suitable for suturing. 

This notion is supported by our findings on collagen content. hAM stroma consists largely of collagen I, II, III, V and VI and thus resembles the structural composition of the cornea and conjunctiva [41]. Accordingly, collagen content is an indicator for the maintenance of tissue integrity of the stroma of cryopreserved hAM. Storage at −28 °C instead of −80 °C showed no significant effect on the concentration of collagen in hAM.

The release of growth factors and cytokines is a key mechanism for improved wound healing and the anti-fibrotic and anti-inflammatory effects in hAM transplantation [31,47,48]. We therefore assayed two particularly relevant growth factors that were found in high concentrations in other studies: HGF and bFGF [31,39]. HGF, along with EGF and KGF, facilitates re-epithelialization and is the one with the highest concentrations found in hAM [31]. bFGF plays an important yet not fully understood role in re-epithelialization and collagen deposition during wound healing [49]. We could see a high variation in growth factor levels in samples from different donor placentas. These results are consistent with previous studies that report high inter-donor variation in growth factor content of hAM [31,39,47,48,50,51]. While preservation at −28 °C caused a slightly higher loss of HGF than cryopreservation at −80 °C in our samples, most hAM stored at −28 °C still contained more HGF than the lowest hAM stored at −80 °C. Hence, it is unclear whether growth factor degradation is likely to have an impact on clinical effects after hAM has been stored at −28 °C. Moreover, this study was able to examine only two growth factors that are highly expressed, while there is a plethora of other soluble factors in hAM that affect regeneration. Further studies on the clinical use of hAM stored under modified conditions are therefore required, particularly for ocular surface diseases where the hAM’s growth factors play an important role.

## 5. Conclusions

Overall, the findings of this study indicate that cryopreservation of amniotic membranes at −28 °C has no overt disadvantages compared to −80 °C as the essential, therapeutically relevant characteristics of hAM are sufficiently preserved. Thus, −28 °C cryopreservation of hAM could be an easy-to-implement storage method, particularly in regions where infrastructure does not allow for storage at −80 °C.

## Figures and Tables

**Table 1:** Donor age and storage time of hAM samples.

Donor	Age in Years	Storage in Months
hAM1	34	7
hAM2	31	12
hAM3	39	11
hAM4	34	8
hAM5	35	7
hAM6	40	7
hAM7	30	7
hAM8	30	7

**Table 2:** Scoring and exemplary HE-stained histological images of hAM epithelium. Scale bar 50 µm.

Score	Criteria	Representative Image
0	- Intact, continuous and regularly shaped epithelium. - No pyknotic, karyorrhectic or karyolytic nuclei.	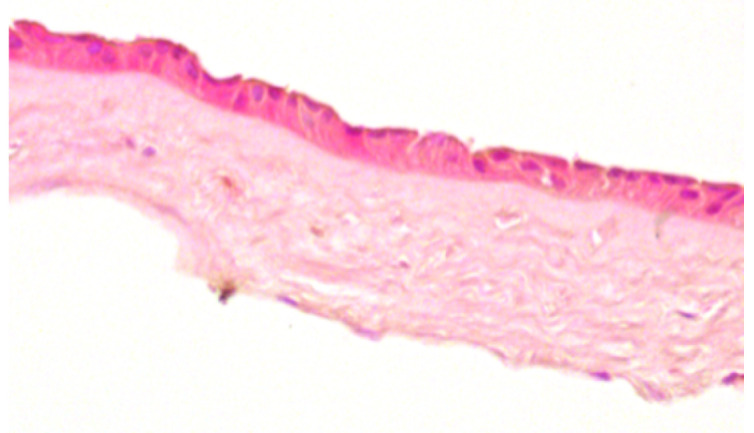
1	- Slightly disarranged cells or small gaps between regular formed epithelial cells. - Few pyknotic, karyorrhectic or karyolytic nuclei.	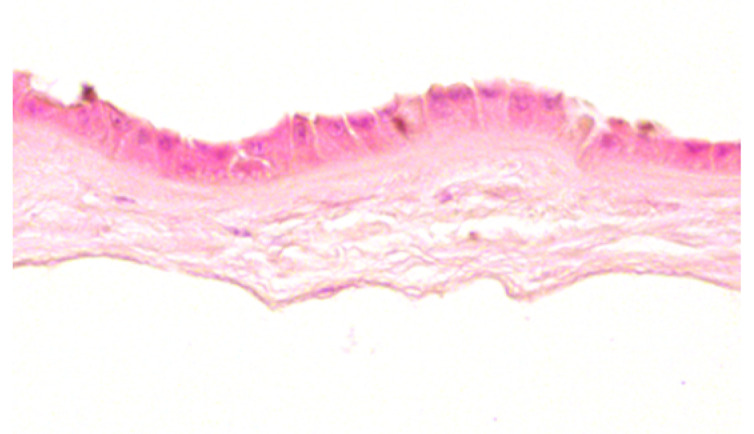
2	- Discontinuously and disarranged epithelium. - Many pyknotic, karyorrhectic or karyolytic nuclei.	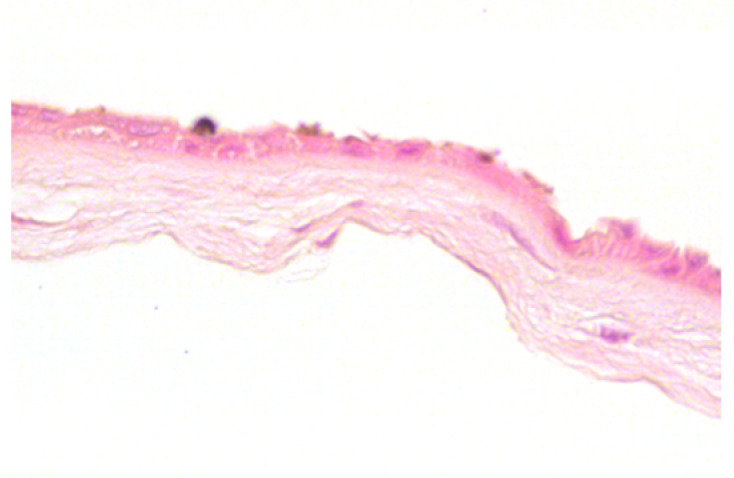
3	- Highly disarranged or missing epithelium with amorph epithelial cells. - Nuclei not visible.	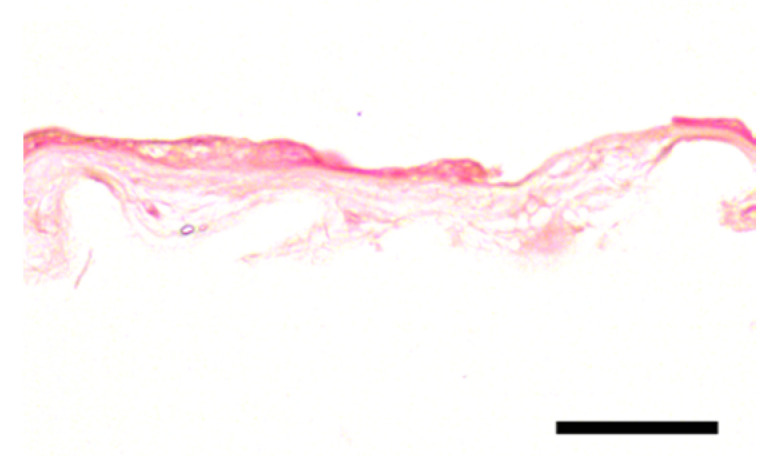

## Data Availability

The datasets generated during the study can be made available from the corresponding author upon request.
